# High Compliance with Scheduled Nimodipine Is Associated with Better Outcome in Aneurysmal Subarachnoid Hemorrhage Patients Cotreated with Heparin Infusion

**DOI:** 10.3389/fneur.2017.00268

**Published:** 2017-06-09

**Authors:** Aaron Wessell, Matthew J. Kole, Neeraj Badjatia, Gunjan Parikh, Jennifer S. Albrecht, David L. Schreibman, J. Marc Simard

**Affiliations:** ^1^Department of Neurosurgery, University of Maryland School of Medicine, Baltimore, MD, United States; ^2^Department of Neurology, University of Maryland School of Medicine, Baltimore, MD, United States; ^3^Department of Anesthesiology, University of Maryland School of Medicine, Baltimore, MD, United States; ^4^Department of Epidemiology and Public Health, University of Maryland School of Medicine, Baltimore, MD, United States; ^5^Department of Pathology, University of Maryland School of Medicine, Baltimore, MD, United States; ^6^Department of Physiology, University of Maryland School of Medicine, Baltimore, MD, United States

**Keywords:** nimodipine, subarachnoid hemorrhage, cerebral aneurysm, cerebral vasospasm, heparin, vascular disorders

## Abstract

**Introduction:**

We sought to determine whether compliance with scheduled nimodipine in subarachnoid hemorrhage patients impacted patient outcomes, with the intent of guiding future nimodipine management in patients who experience nimodipine-induced hypotension.

**Methods:**

We performed a retrospective analysis of 118 consecutive aneurysmal subarachnoid hemorrhage patients treated with the Maryland Low-Dose IV Heparin Infusion Protocol. Patients were categorized into three independent nimodipine compliance groups: ≥1 dose held, ≥1 dose split, and no missed or split-doses. A split-dose was defined as 30 mg of nimodipine administered every 2 h. Our primary outcome was discharge to home. Bivariate and multivariable logistic regression analyses were used to assess predictors of discharge disposition as a function of nimodipine compliance.

**Results:**

Of the 118 patients, 20 (17%) received all nimodipine doses, 6 (5%) received split-doses but never had a full dose held, and 92 (78%) had ≥1 dose held. Forty-five percent of patients were discharged to home, including 75% who received all doses, 67% who received ≥1 split-doses, and 37% with ≥1 missed doses (*p* = 0.003). Multivariable analysis showed that, along with age and World Federation of Neurosurgical Societies grade, nimodipine compliance was an independent predictor of clinical outcome; compared to missing one or more nimodipine doses, full dosing compliance was associated with increased odds of discharge to home (odds ratio 5.20; 95% confidence intervals 1.46–18.56).

**Conclusion:**

In aneurysmal subarachnoid hemorrhage patients with modified Fisher scores 2 through 4 who are cotreated with a low-dose heparin infusion, full compliance with nimodipine dosing was associated with increased odds of discharge to home.

## Introduction

Aneurysmal subarachnoid hemorrhage is associated with substantial morbidity and mortality (1). Approximately one-third of those who survive the initial hemorrhage remain dependent, while another 20–30% who present with a low clinical grade will experience further decline from their admission neurologic status (1, 2). For those who survive the ictus, the onset of delayed neurological deficit (DND), occurring in nearly 30% of patients, is the most important factor impacting patient outcome (3, 4). DND refers collectively to the neurological, psychological, and cognitive effects experienced by those who have suffered a subarachnoid hemorrhage. The mechanisms implicated in DND include arterial vasospasm, microthrombosis, oxidative damage, and neuroinflammation, with resultant cerebral infarction, demyelination, cerebral edema, endothelial, and neuronal cell death (5–14).

Nimodipine is the only pharmacological agent that has been consistently shown in randomized, placebo-controlled trials to improve outcomes in aneurysmal subarachnoid hemorrhage (15–19). Nimodipine is a lipophilic dihydropyridine calcium-channel blocker that crosses the blood–brain barrier and prevents the influx of extracellular calcium into vascular smooth muscle, neuronal, and other cells (20). Nimodipine may exhibit a degree of selectivity for the cerebral vasculature due to its greater dependence on extracellular calcium for smooth muscle contraction. However, hypotension, along with other adverse systemic effects such as edema, abnormal liver function, headache, flushing, and gastrointestinal symptoms, have been reported with nimodipine use (15–19).

A significant percentage of patients admitted with aneurysmal subarachnoid hemorrhage demonstrate sensitivity to nimodipine’s hypotensive effect. This presents a clinical dilemma that served as the impetus for the present study—should nimodipine dosing be reduced or stopped in the face of drug-induced hypotension, or should dosing be maintained, with vasopressors used as required to counteract hypotension? Patients who cannot tolerate nimodipine will not benefit from its neuroprotectant, anti-inflammatory, and pro-fibrinolytic effects (21–33), and consequently may have poorer outcomes after subarachnoid hemorrhage.

The purpose of this study was to assess the association between nimodipine dosing compliance and clinical outcome at time of discharge after aneurysmal subarachnoid hemorrhage in patients cotreated with a continuous low-dose heparin infusion.

## Materials and Methods

### Patient Selection

The present study was conducted with approval of the University of Maryland Medical Center IRB. The records of all patients treated for aneurysmal subarachnoid hemorrhage by a single cerebrovascular neurosurgeon (J. Marc Simard) between July 1, 2008 and July 1, 2015 were reviewed. Patients underwent surgical clipping, endovascular coiling, or a combination of the two. All patients underwent treatment with the Maryland Low-Dose IV Heparin Infusion Protocol, consisting of 12 U/kg/h of unfractionated heparin, administered by constant IV infusion, beginning 12 h after aneurysm treatment, which is a practice pattern of the senior author (34–36). Patients with modified Fisher scores of 2 through 4 were included, as this group is at greatest risk of developing cerebral vasospasm. Those with insufficient hospital and medication-administration records were excluded from analysis. None of the patients in the present cohort were included in a previous retrospective analysis by Simard et al. published in 2013 (36).

### Management of Aneurysmal Subarachnoid Hemorrhage Patients

All patients were treated according to the American Heart Association’s Guidelines for the Management of Aneurysmal Subarachnoid Hemorrhage (37, 38), with an emphasis on prompt aneurysm obliteration, drainage of cerebrospinal fluid for treatment of hydrocephalus, maintenance of euvolemia, and administration of oral nimodipine. Radiographic studies, such as computed tomography (CT), CT angiogram (CTA), digital subtraction angiogram (DSA), and/or magnetic resonance angiography, were obtained routinely within 24 h of treatment to confirm obliteration of the aneurysm, rule out any complication of treatment, and evaluate for the development of hydrocephalus.

If patients experienced neurological deterioration attributed to vasospasm, regardless of radiographic confirmation, patients were treated with hemodynamic augmentation consisting of IV fluid bolus to assure euvolemia and vasopressor-induced hypertensive therapy with phenylephrine or norepinephrine to increase systolic blood pressure by 20–40%. The decision to obtain vascular imaging for evaluation of vasospasm, such as a CTA or DSA, was at the discretion of the attending neurointensivist. Daily monitoring for vasospasm with transcranial Doppler or electroencephalogram was not routinely performed. If clinical deterioration persisted despite hemodynamic augmentation, and radiographic study confirmed the presence of vasospasm, selective catheter angiography was performed with administration of intra-arterial calcium-channel blocker. Angioplasty was performed at the discretion of the attending neuro-interventional radiologist in select cases when intra-arterial vasodilator therapy did not produce a sufficient radiographic improvement in blood vessel caliber and/or arterial filling as observed on catheter cerebral angiogram.

### Nimodipine Use in Subarachnoid Hemorrhage

Patients were treated with 60 mg of oral nimodipine every 4 h beginning within 96 h of the ictus according to the AHA guidelines (37). At our institution, patients are initiated on oral nimodipine at the time of admission and are maintained on this medication until discharge, up to a maximum of 21 days. The onset of hypotension (systolic blood pressure <90 mm Hg) following administration of nimodipine, regardless of whether or not a patient was actively undergoing treatment with vasopressor-induced hypertension, may have prompted a change in dosing to 30 mg every 2 h. Alternatively, a given dose may have been withheld if the hypotension was lasting, associated with a change in neurological examination, or associated with reoccurring blood pressure drops in the setting of active vasospasm requiring frequent rescue therapy with vasopressors. The decision to do so was based on individual patient characteristics, as determined by the attending neurointensivist.

### The Maryland Low-Dose IV Heparin Infusion Protocol

The Maryland Low-Dose IV Heparin Infusion Protocol for use in aneurysmal subarachnoid hemorrhage has been described previously (36). In the original report, use of a low-dose heparin infusion for approximately 14 days after aneurysm treatment was associated with a reduced incidence of DND, requirement for rescue therapy, cerebral infarction, and discharge to inpatient rehabilitation (36). Heparin is a pleiotropic drug that may antagonize several biomolecular pathways implicated in delayed neurologic decline following aneurysmal subarachnoid hemorrhage. Unfractionated heparin complexes with oxyhemoglobin, blocks the activity of free radicals, antagonizes endothelin-mediated vasoconstriction, smooth muscle depolarization, and inflammation (34, 35). All of the patients included in the present analysis underwent treatment with a low-dose heparin infusion. On post-bleed day 14, a screening CTA with perfusion analysis was performed to assess for radiographic vasospasm. If there was only mild or no vasospasm, the heparin infusion was discontinued and in the patient was considered for downgrading to a lower level of care if they did not have a ventriculostomy catheter in place. In select cases of low World Federation of Neurosurgical Societies (WFNS) grade subarachnoid hemorrhage, if the patient had not suffered any delayed neurologic decline and had a stable neurologic examination, an early screening CTA (at post-bleed day 10, for example) was performed and the patient was then downgraded to a lower level of care as deemed appropriate by the ICU provider.

### Data Compilation

Patient records, including hospital records, operative reports, clinic notes, and radiographic studies, were reviewed. Demographic, clinical, radiographic, and outcomes data were compiled.

The occurrence of angiographic vasospasm was determined from review of CTA and DSA studies by two neurosurgeons and independently by neuroradiologists from the University of Maryland. Studies demonstrating angiographic vasospasm in any vascular territory in comparison to a baseline study were characterized as mild (0–33%), moderate (34–66%), or severe (67–100%) vessel narrowing, independent of the patient’s clinical/neurologic status (39). The incidence of DND was determined from review of the patient’s neurologic status as documented in hospital records. The presence of angiographic vasospasm in a patient with neurological deterioration was deemed clinical vasospasm, or a delayed neurologic deficit. DND was further defined to include any neurologic decline, such as a change in mental status, pronator drift, or focal neurologic deficit without any other attributable medical causes regardless of radiographic confirmation of vasospasm.

The occurrence of new ischemia-related CT hypodensities was determined from review of all CT scans by two neurosurgeons and independently by neuroradiologists from the University of Maryland. CT scans obtained during hospitalization and on follow-up were correlated with CTA or DSA and neurological examination to determine whether new hypodensities were attributable to vasospasm (within a discrete vascular or watershed territory), as opposed to resulting from intraparenchymal hemorrhage, intraventricular catheter placement, surgical retraction, or surgery-related loss of a perforating artery.

### Exposure Definition

We created three independent groups based on compliance with nimodipine dosing during hospitalization. Nimodipine administration was evaluated by review of medication-administration records. Blood pressure data were obtained from review of nursing flow sheets recorded during the first 21 days of hospitalization following aneurysm rupture. Nimodipine doses held for relative hypotension, irrespective of whether or not a patient was actively undergoing treatment with vasopressor-induced hypertension, were recorded. Nimodipine administered as 30 mg every 2 h was considered a split-dose. A patient who required a split-dose but later had a dose held was grouped among patients who missed one or more complete doses. Patients receiving all scheduled doses, with no split-doses received, were the final group. A small number of patients (*n* = 5, 4%) never received Nimodipine due to significant hemodynamic instability on admission or cardiac dysfunction and were grouped with the doses held group. Dosing alterations during surgical or endovascular treatment were not considered in the analysis.

### Outcome Definition

We defined a good outcome as discharge to home. Discharge disposition previously has been reported as an outcome measure in studies of subarachnoid hemorrhage, serving as a surrogate for short-term functional outcome (40, 41). While several non-clinical factors may influence one’s discharge disposition, past reports have shown that discharge status correlates with modified Rankin Scale score and functional outcome at 90 days in stroke patients (40, 42). Recommendations for discharge to home versus inpatient rehabilitation or nursing facility were made independently by physical and/or occupational therapists according to activities of daily living based on the patient’s degree of functional independence. All other discharge locations, including in-hospital mortality, comprised “discharge to other.” Glasgow Outcomes Scale (GOS) scores at time of discharge were calculated in a retrospective manner from clinical documentation.

We assessed the distribution and frequency of all variables. Covariates were compared between nimodipine compliance categories using the Fisher–Freeman–Halton test, ANOVA, or the Kruskal–Wallis test. Covariates were compared between outcome groups using Chi-Square Goodness of Fit, Student’s *t*-test, or the Wilcoxon rank sum test. We assessed the unadjusted association between nimodipine compliance categories and discharge to home using logistic regression. Covariates that were associated with the exposure and outcome but not in the causal pathway (i.e., did not occur after initial nimodipine exposure) were considered for inclusion in our final logistic regression model (43, 44). We assessed potential effect modification *a priori* by age and WFNS grade by creating interaction terms in the logistic regression model. Odds ratios (OR) and 95% confidence intervals (CI) are reported.

Due to the small number of patients who received a split-dose of nimodipine but did not miss any doses, we conducted sensitivity analyses to assess whether inclusion of these individuals with the other compliance groups would change effect estimates. All analyses were conducted using SAS version 9.3 (SAS Institute, Cary, NC, USA). A *p*-value <0.05 was considered significant.

## Results

### Demographic Characteristics

Data from 118 consecutive patients were analyzed, all of whom were treated with the Maryland Low-Dose IV Heparin Infusion Protocol (36). The demographic characteristics of patients, grouped according to their nimodipine compliance, are shown in Table 1. Age, sex, medical history, WFNS grade, GCS motor score, and modified Fisher score did not differ significantly among compliance groups. Anterior cerebral artery aneurysms were the most common (45.8%). The majority of patients underwent treatment of their aneurysm within 20 h of admission, with 108 patients undergoing surgical clipping and 10 undergoing endovascular coiling. 11 (10.2%) patients had an intraparenchymal hematoma evacuated during open surgery. Admission echocardiograms were performed on 45 (38%) patients with a mean ejection fraction of 60.87% (SD, 13.63).

**Table 1:** Characteristics of patients with subarachnoid hemorrhage from brain aneurysm rupture by nimodipine compliance, *n* = 118.

Characteristic	Full nimodipine compliance, *n* = 20	At least one nimodipine dose split, *n* = 6	At least one nimodipine dose held, *n* = 92	*p*-Value^a^
Age, mean (SD)	51.4 (10.3)	58.3 (9.9)	56.5 (13.0)	0.23
Female, *n* (%)	14 (70)	4 (67)	59 (64)	0.93
History of tobacco use, *n* (%)	11 (55)	2 (33)	51 (55)	0.64
History of hypertension, *n* (%)	12 (60)	4 (67)	57 (62)	>0.99
History of statin use, *n* (%)	2 (10)	0	6 (7)	0.76
History of cocaine use, *n* (%)	0	0	3 (3)	>0.99
History of marijuana use, *n* (%)	2 (10)	0	6 (7)	0.76
WFNS (admission), *n* (%)				0.17
1	8 (40)	4 (67)	19 (21)	
2	4 (20)	0	30 (33)	
3	0	0	1 (1)	
4	7 (35)	1 (17)	33 (36)	
5	1 (5)	1 (17)	9 (10)	
GCS motor score, *n* (%)				0.74
6	16 (80)	4 (67)	61 (66)	
5	3 (15)	1 (17)	21 (23)	
4	1 (5)	0	5 (5)	
<4	0	1 (17)	5 (5)	
MFS (admission), *n* (%)				0.39
2	3 (15)	1 (17)	5 (5)	
3	14 (70)	4 (67)	70 (76)	
4	3 (15)	1 (17)	17 (18)	
Aneurysm location, *n* (%)^b^				0.69
Internal carotid artery	6 (30)	2 (33)	23 (26)	
Anterior cerebral artery	6 (30)	3 (50)	45 (49)	
Middle cerebral artery	7 (35)	1 (17)	18 (20)	
Posterior circulation	1 (5)	0	6 (7)	
Procedure type, *n* (%)				0.82
Clip	19 (95)	6 (100)	83 (90)	
Coil	1 (5)	0	9 (10)	
>20 h to surgery, *n* (%)	7 (35)	2 (33)	35 (38)	>99.0
Vasospasm severity, *n* (%)				0.04
none	11 (55)	3 (50)	25 (27)	
mild	0	2 (33)	20 (22)	
moderate	5 (25)	0	25 (27)	
severe	4 (20)	1 (17)	22 (24)	
VIH, *n* (%)	3 (15)	3 (50)	46 (50)	0.01
Systolic blood pressure, mean (SD)	139.2 (15.5)	148.1 (16.4)	145.2 (20.5)	0.41
Days on pressors, mean (SD)	0.3 (0.8)	5.2 (6.5)	2.9 (4.2)	0.008
IAT needed, *n* (%)	0	1 (17)	22 (24)	0.03
Angioplasty, *n* (%)	0	0	6 (7)	0.70
DND, *n* (%)	4 (20)	3 (50)	48 (52)	0.03
CT infarction, *n* (%)	1 (5)	1 (17)	12 (13)	0.45
Shunt inserted, *n* (%)	2 (10)	2 (33)	27 (29)	0.17
ICU length of stay, mean (SD)	15.7 (7.0)	25.3 (18.5)	19.9 (8.5)	0.04
GOS > 3, *n* (%)	19 (95)	5 (83)	65 (71)	0.05
Discharged home, *n* (%)	15 (75)	4 (67)	34 (37)	0.003
Died, *n* (%)	0	0	7 (8)	0.55

*^a^p-Values from the Fisher–Freeman–Halton test, ANOVA, or the Kruskal–Wallis test*.

*WFNS, World Federation of Neurosurgical Societies Scale; GCS, Glasgow Coma Scale; MFS, Modified Fisher Scale; DND, delayed neurological deficit; VIH, vassopressor-induced hypertension; IAT, intra-arterial treatment; ICU, intensive care unit; GOS, Glasgow Outcomes Scale*.

*^b^Aneurysms by location: anterior cerebral artery (A1, anterior communicating artery, A2 and pericallosal aneurysms); middle cerebral artery; internal carotid artery (anterior choroidal artery, posterior communicating artery, and carotid terminus aneurysms); posterior circulation (vertebral artery, basilar artery, posterior inferior cerebellar artery, anterior inferior cerebellar artery and superior cerebellar artery aneurysms)*.

### Nimodipine Use

Twenty patients (17%) received 100% of the scheduled nimodipine doses from the time of admission through the end of their treatment period, 6 patients (5%) received at least one split-dose, and 92 (78%) missed one or more doses.

### Vasospasm

A total of 79 patients (67%) were found to have angiographic vasospasm on CTA or DSA, with 27 (23%) of those cases being severe. As shown in Table 1, vasospasm severity (*p* = 0.04), DND (*p* = 0.03), use of vasopressor-induced hypertension (*p* = 0.01), days on vasopressors (*p* = 0.008), and the use of intra-arterial therapy (*p* = 0.003) differed significantly among compliance groups. There was no significant difference in the use of angioplasty or systolic blood pressure.

### Outcomes

Overall, patients suffered a very low rate of CT infarction, with an incidence of 12%. As shown in Table 1, the incidence of CT infarction, shunt insertion, and death did not differ significantly between compliance groups. Patients with full compliance were more likely to be discharged home (75%), compared to those who received one or more split nimodipine doses (67%) or those who missed one or more full-doses (37%) (*p* = 0.003). Similarly, patients with full compliance tended to have a shorter ICU stay (*p* = 0.04) and GOS > 3 at discharge (95%) compared to those who received one or more split-doses (83%) or those who missed one or more full-doses (71%) (*p* = 0.05). All patients who died missed one or more nimodipine doses.

### Discharge Disposition

The characteristics of patients grouped by discharge disposition are shown in Table 2. Fifty-three patients (45%) were discharged to home and 65 (55%) were discharged to other locations, including 7 who died. There were no readmissions for late DND after discharge. In bivariate analysis, age (*p* < 0.001), WFNS grade (*p* < 0.001), admission GCS motor score (*p* = 0.003), and modified Fisher score (*p* = 0.002) were significantly associated with discharge disposition, whereas the only medical history that was significantly associated was hypertension (*p* = 0.03).

**Table 2:** Characteristics of patients with subarachnoid hemorrhage from brain aneurysm rupture 2008–2015 by discharge to home, *n* = 118.

Characteristic	Discharge home, *n* = 53	Discharge to Other location, *n* = 65	*p*-Value^a^
Age, mean (SD)	51.3 (11.1)	59.4 (12.4)	<0.001
Female, *n* (%)	34 (64.2)	43 (66.2)	0.82
History of tobacco use, *n* (%)	25 (47.2)	39 (60.0)	0.16
History of hypertension, *n* (%)	27 (50.9)	46 (70.8)	0.03
History of statin use, *n* (%)	3 (5.7)	5 (7.7)	0.66
History of cocaine use, *n* (%)	1 (1.9)	2 (3.1)	>0.99
History of marijuana use, *n* (%)	4 (7.6)	4 (6.2)	>0.99
WFNS, *n* (%)			<0.001
1	21 (39.6)	10 (15.4)	
2	20 (37.7)	14 (21.5)	
3	0	1 (1.5)	
4	10 (18.9)	31 (47.7)	
5	2 (3.8)	9 (13.9)	
GCS motor score, *n* (%)			0.003
6	46 (87)	35 (54)	
5	5 (9)	20 (31)	
4	2 (4)	4 (6)	
<4	0	6 (9)	
MFS, *n* (%)			0.002
2	5 (9.4)	4 (6.2)	
3	46 (86.8)	42 (64.6)	
4	2 (3.8)	19 (29.2)	
Aneurysm location, *n* (%)^b^			0.39
Internal carotid artery	12 (23)	19 (29)	
Anterior cerebral artery	22 (42)	32 (49)	
Middle cerebral artery	15 (28)	11 (17)	
Posterior circulation	4 (8)	3 (5)	
Procedure type, *n* (%)			0.18
Clip	51 (96.2)	57 (87.7)	
Coil	2 (3.8)	8 (12.3)	
>20 h to surgery, *n* (%)	18 (34.0)	26 (40.0)	0.50
Nimodipine compliance, *n* (%)		5 (8)	0.18
All doses received	15 (28)	5 (8)	
At least one dose split	4 (8)	2 (3)	
At least one dose missed	34 (64)	58 (89)	
Vasospasm severity, *n* (%)			0.05
none	21 (39.6)	18 (27.9)	
mild	12 (22.6)	10 (15.4)	
moderate	14 (26.4)	16 (24.6)	
severe	6 (11.3)	21 (32.3)	
VIH, *n* (%)	12 (22.6)	40 (61.5)	<0.001
Systolic blood pressure, mean (SD)	134.5 (20.0)	152.4 (15.3)	<0.001
Days on pressors, M (IQR)	0 (0,0)	2.0 (0, 6.0)	<0.001
IAT needed, *n* (%)	3 (5.7)	20 (30.8)	<0.001
Angioplasty, *n* (%)	0	6 (9.2)	0.02
DND, *n* (%)	12 (22.6)	43 (66.2)	<0.001
CTinfarction, *n* (%)	1 (1.9)	13 (20.0)	0.003
Shunt inserted, *n* (%)	9 (17.0)	22 (33.9)	0.04
ICU length of stay, mean (SD)	15.5 (5.4)	22.8 (10.2)	<0.001
GOS > 3, *n* (%)	53 (100)	36 (55.4)	<0.001
Died, *n* (%)	0	7 (10.8)	0.01

*^a^p-Value from Chi-square Goodness of Fit, Student’s t-test, or Wilcoxon rank sum*.

*WFNS, World Federation of Neurosurgical Societies Scale; GCS, Glasgow Coma Scale; MFS, Modified Fisher Scale; DND, delayed neurological deficit; VIH, vassopressor-induced hypertension; IAT, intra-arterial treatment; ICU, intensive care unit; GOS, Glasgow Outcomes Scale*.

*^b^Aneurysms by location: anterior cerebral artery (A1, anterior communicating artery, A2 and pericallosal aneurysms); middle cerebral artery; internal carotid artery (anterior choroidal artery, posterior communicating artery, and carotid terminus aneurysms); posterior circulation (vertebral artery, basilar artery, posterior inferior cerebellar artery, anterior inferior cerebellar artery and superior cerebellar artery aneurysms)*.

Nimodipine dosing compliance was significantly associated with discharge disposition (*p* = 0.003). Also, vasopressor-induced hypertension, systolic blood pressure, days on vasopressors, and the use of intra-arterial therapy were significantly associated with discharge disposition (all *p* < 0.001). In addition, DND, CT infarction, shunt insertion, and ICU length of stay were significantly associated with discharge disposition (*p* values ranging from 0.04 to <0.001).

Prior to adjustment for confounding, we found that full nimodipine dosing compliance was significantly associated with discharge to home (OR, 5.12; 95% CI, 1.71–15.33) compared to missing at least one dose. Receiving at least one split-dose but not missing any doses also was associated with discharge to home (OR, 3.41; 95% CI, 0.59–19.62), but this association was not statistically significant.

Our final logistic regression model included an indicator for the nimodipine compliance categories, age, and a WFNS grade indicator (3 and 4 were grouped due to small cell sizes) (Table 3). There was no effect modification by age or WFNS grade. Full nimodipine dosing compliance was significantly associated with discharge to home (OR, 5.20; 95% CI, 1.46–18.56). In addition, greater age (OR, 0.95; 95% CI, 0.91–0.98), and WFNS grades 3/4 (OR, 0.16; 95% CI, 0.05–0.60) or 5 (OR, 0.12; 95% CI, 0.02–0.70) were significantly associated with poor discharge disposition. Receiving a split-dose of nimodipine (30 mg every 2 h) was not significantly associated with a worse outcome. Sensitivity analyses revealed that varying groupings by receiving a split-dose of nimodipine had no impact on effect estimates (Table 4).

**Table 3:** Adjusted odds ratios of Discharge to Home Among Patients with Subarachnoid Hemorrhage from Brain Aneurysm Rupture 2008–2015, *n* = 118.

	Odds ratio (95% confidence interval)
**Unadjusted association**	
Nimodipine compliance	
At least one dose held	Reference
At least one dose split	3.41 (0.59, 19.62)
All doses received	5.12 (1.71, 15.33)
**Adjusted association**	
Nimodipine compliance	
At least one dose held	Reference
At least one dose split	3.99 (0.54, 29.60)
All doses received	5.20 (1.46, 18.56)
Age, per year	0.95 (0.91, 0.98)
WFNS	
1	Reference
2	0.89 (0.28, 2.79)
3, 4	0.16 (0.05, 0.50)
5	0.12 (0.02, 0.70)

*WFNS, World Federation of Neurosurgical Societies Scale*.

**Table 4:** Sensitivity analyses.^a^

	Odds ratio (95% confidence interval)
**Nimodipine compliance**	
At least one dose held	Reference
All doses received or at least one split	4.86 (1.58, 14.93)
**Nimodipine compliance**	
At least one dose split or held	Reference
All doses received	4.71 (1.33, 16.63)

*^a^Adjusted for recoded World Federation of Neurosurgical Societies Scale and age*.

## Discussion

Nimodipine is the only pharmacologic agent shown in randomized placebo-controlled trials to improve outcome (15–19). Despite its widespread use, there are little data to guide the management of hypotension following nimodipine administration. The original randomized-controlled trial of nimodipine by Allen et al. (15) did not report any hypotensive effects. Subsequent studies comprised of patients with higher clinical grades reported various adverse effects of nimodipine, most commonly hypotension, leading to early cessation of the medication (16–19). In our study population, 45% of patients had a WFNS grade of III or higher, and 78% of patients experienced hypotension requiring that one or more doses be held.

The Neurocritical Care Society’s Multidisciplinary Consensus Conference states that if nimodipine causes hypotension, then dosing should be lowered and administered at more frequent intervals (38). Alternative dosing options include splitting the nimodipine dose into a 30 mg dose given every 2 h, halving the nimodipine dose by giving 30 mg every 4 h, holding nimodipine altogether, or administering volume resuscitation or vasopressor agents to counteract the hypotensive effects. Our institutional practice is that, if patients develop hypotension, dosing of nimodipine is changed to 30 mg every 2 h, and if patients continue to experience hypotension, nimodipine may be held altogether.

We evaluated the predictors of discharge to home after admission for aneurysmal subarachnoid hemorrhage, to determine the impact of nimodipine compliance on outcomes at the time of discharge. Our study demonstrates that, along with age and WFNS grade, compliance with scheduled nimodipine is an independent predictor of clinical outcome. We found that patients who missed one or more doses of nimodipine experienced a significantly worse clinical outcome. The recent study by Sandow et al. (45) compared outcomes in patients who received all doses of nimodipine to those who received 30 mg every 4 h or underwent early cessation of nimodipine due to its hypotensive effects. They found that only 43.6% of patients completed a full 14-day course of nimodipine without dose reduction or early cessation. Patients with dose reductions or early cessation experienced greater rates of angiographic vasospasm and cerebral ischemia and were found to have unfavorable clinical outcomes on multivariate analysis.

Various systemic effects, primarily catecholamine-mediated, have been described in association with subarachnoid hemorrhage (46). However, a link between these physiologic derangements and an increased sensitivity to nimodipine remains unproven. While patients with higher grades of subarachnoid hemorrhage may have a greater impairment of cerebral autoregulatory capacity in the days following the ictus, a clear explanation for a greater sensitivity to nimodipine’s hypotensive effect is lacking (47–51). Many patients with high Hunt and Hess subarachnoid hemorrhage exhibit increased cardiac troponin I release, regional wall motion abnormalities on echocardiogram, and are more likely to require vasopressor/inotropic infusions (52). Reversible neurogenic myocardial “stunning” related to aneurysmal subarachnoid hemorrhage leads to an increased propensity to delayed ischemic neurologic deterioration related to vasospasm (53), theoretically making compliance with the administration of nimodipine more important.

Although many of our findings are consistent with those of Sandow et al. (45), there were some differences. Some of our patients received 30 mg every 2 h, whereas patients in the Sandow et al. study received doses of 30 mg every 4 h. Sandow et al. determined that the vasopressor dose during the first 14 days was an independent predictor of outcome on multivariable analysis, while we did not include this parameter in our final logistic regression model. Perhaps most notable, our patient’s experienced lower rates of cerebral infarction across all dosing groups, which may be attributed to unknown patient characteristics or, as previously reported (36), to the use of a continuous low-dose heparin infusion in our patients.

This study has several limitations, including its retrospective nature and modest sample size. While we stratified patients based on the clinical and radiographic severity of hemorrhage, our classification of patients may not fully account for unmeasured markers of severity, such as concomitant cardiac dysfunction or infection/sepsis, which may confound outcomes data. The study of patients who were all treated with a low-dose IV heparin infusion may limit the generalizability of our findings. Causality remains enigmatic—it is not known whether forcing full compliance would yield more favorable outcomes, or whether patients intolerant to nimodipine, regardless of treatment, are destined to poor outcomes. Regardless, our results are consistent with recent reports that highlight the negative effects of missing nimodipine doses (45). Prospective, randomized-controlled trials will be required to advance understanding of the effects of nimodipine compliance and of combined therapeutic agents in subarachnoid hemorrhage.

## Conclusion

Nimodipine is the only agent that has consistently been shown to improve outcomes in patients with aneurysmal subarachnoid hemorrhage. From a practical standpoint, however, the hypotensive effect of nimodipine limits compliance with scheduled dosing. Our study reinforces the importance of nimodipine compliance and its effect on outcomes in aneurysmal subarachnoid hemorrhage, as it was one of three statistically significant predictors of discharge to home.

## Ethics Statement

This study was conducted under the approval of the Institutional Review Board at the University of Maryland School of Medicine. All procedures performed involving human participants were in accordance with the ethical standards of the institutional and/or national research committee and with the 1964 Helsinki declaration and its later amendments or comparable ethical standards.

## Author Contributions

All authors made substantial contributions to the following: study design; data acquisition, analysis, and interpretation; manuscript preparation and final manuscript approval.

## Conflict of Interest Statement

AW, MK, NB, GP, and DS have no disclosures or conflicts of interest. JA has no conflicts of interest. She is supported by an AHRQ grant 1K01HS024560. JS has no disclosures or conflicts of interest. He is supported by grants from the NINDS (NS060801) and the Department of Veterans Affairs (BX001629; BX002889). This research was conducted in the absence of any commercial or financial relationships that could be construed as potential conflicts of interest.
